# Detection of Crimean–Congo haemorrhagic fever virus in *Hyalomma marginatum* ticks, southern France, May 2022 and April 2023

**DOI:** 10.2807/1560-7917.ES.2024.29.6.2400023

**Published:** 2024-02-08

**Authors:** Célia Bernard, Charlotte Joly Kukla, Ignace Rakotoarivony, Maxime Duhayon, Frédéric Stachurski, Karine Huber, Carla Giupponi, Iyonna Zortman, Philippe Holzmuller, Thomas Pollet, Mélanie Jeanneau, Alice Mercey, Nathalie Vachiery, Thierry Lefrançois, Claire Garros, Vincent Michaud, Loic Comtet, Léa Despois, Philippe Pourquier, Caroline Picard, Alexandra Journeaux, Damien Thomas, Sabine Godard, Elodie Moissonnier, Stéphane Mely, Manon Sega, Delphine Pannetier, Sylvain Baize, Laurence Vial

**Affiliations:** 1 Centre de coopération internationale en recherche agronomique pour le développement (CIRAD), University of Montpellier (UMR) Animal Santé Territoires Risques Écosystèmes (ASTRE), Montpellier, France; 2 ASTRE UMR, CIRAD, Institut national de la recherche agronomique (INRAE), Montpellier, France; 3 National Reference Center for Viral Hemorrhagic Fevers, Lyon, France; 4 Unité de Biologie des Infections Virales Emergentes, Institut Pasteur - Centre International de Recherche en Infectiologie (CIRI), Université de Lyon, Institut national de la santé et de la recherche médicale (INSERM) U1111, Ecole Normale Supérieure de Lyon, Université Lyon 1, CNRS UMR5308, Lyon, France; 5 Laboratoire P4 INSERM Jean Mérieux, INSERM Lyon, France; 6 Innovative Diagnostics, Grabels, France; 7 Agence nationale de sécurité sanitaire de l'alimentation, de l'environnement et du travail (ANSES), INRAE, Ecole Nationale Vétérinaire d’Alfort, UMR BIPAR, Laboratoire de Santé Animale, Maisons-Alfort, France; 8 CIRAD, DG, Paris, France

**Keywords:** CCHFV, *Hyalomma marginatum*, cattle, ticks, France

## Abstract

Crimean–Congo haemorrhagic fever (CCHF), a potentially severe zoonotic viral disease causing fever and haemorrhagic manifestations in humans. As the Crimean–Congo haemorrhagic fever virus (CCHFV) has been detected in ticks in Spain and antibodies against the virus in ruminant sera in Corsica, it was necessary to know more about the situation in France. In 2022–2023, CCHFV was detected in 155 ticks collected from horses and cattle in southern France.

The emergence and spread of the tick-borne viral disease Crimean–Congo haemorrhagic fever (CCHF) pose significant challenges to public health. Transmission of the virus to humans occurs predominantly via bites of *Hyalomma* ticks, in Europe especially the species *H. marginatum* and *H. lusitanicum,* or via exposure to infected blood or tissues of viraemic animals or humans [1-3]. Here we describe the detection of Crimean–Congo haemorrhagic fever virus (CCHFV) from ticks in southern France.

## Risk-based sampling

**Figure 1:**
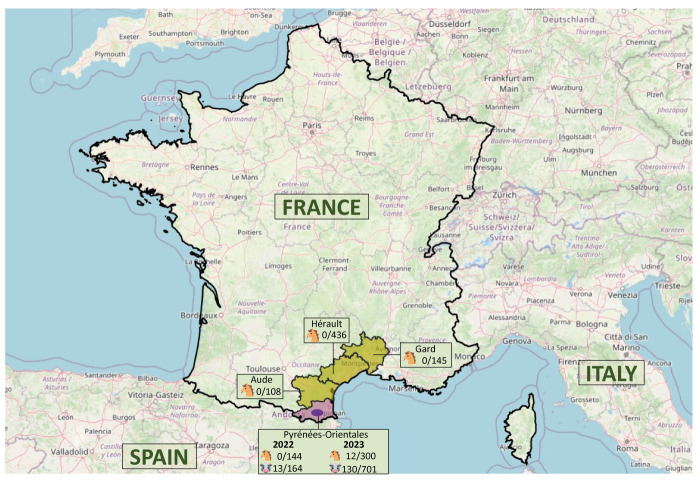
Map showing areas where ticks were collected from cattle and horse farms for analysis of Crimean–Congo haemorrhagic fever virus, France, May 2022 and April 2023 (n =57)

As ticks are the only known natural reservoirs of CCHFV [4], we focused on field collection of ticks. In May 2022, during the peak activity of ticks, we collected ticks mainly from horses in four Mediterranean departments (NUTS-3) on the French mainland bordering Spain (Figure 1). Horses are the likely hosts of adult stages of *H. marginatum* [5,6].

Boxes indicate the name of each department, the animal species (horse or cattle) from which ticks were collected and the number of ticks tested positive for Crimean–Congo haemorrhagic fever virus. The departments coloured green were sampled in 2022 and the one coloured purple was sampled in 2022 and 2023.

Cattle are considered good amplifiers of CCHFV and thus enhance local virus circulation [7]. In 2023, we optimised the sampling by collecting ticks from cattle in the Pyrénées-Orientales department (Figure 1) where antibodies against CCHFV were identified in 2021-2022 from cattle (maps showed in https://www.anses.fr/fr/system/files/SABA2020SA0039Ra.pdf). Cattle farms with the highest within-herd seroprevalences were selected, as well as a few seronegative farms in the same zones. In addition, farms with horses (not previously tested with serology), located in the neighbourhood of the seropositive cattle farms were also visited, especially when the cattle farmers did not give their consent to sampling. As *Hyalomma* ticks are not located within cattle barns but likely in the natural farm environments, specific pastures (e.g. shrublands) were selected. In spring, cattle from different farms are gathered and grazed in such shrublands, and these spring pastures were thus considered as suitable sites for collection of *H. marginatum* [8]. Ticks were collected in April when adult *H. marginatum* search for hosts and potentially infect naïve cattle.

## Laboratory investigations

Ticks were species identified morphologically by experimented acarologists using relevant identification keys [9,10], and only *H. marginatum* ticks were included in the further analysis and stored at -80°C.

After being washed in bleach for 30 s and rinsed three times in water, the ticks were crushed individually in 400 µL of Dulbecco’s Modified Eagle Medium (DMEM) (Eurobio Scientific, Les Ulis, France) supplemented with 10% fetal calf serum. Total RNA was extracted using a kit, NucleoMag VET extraction kit (Macherey-Nagel, Düren, Germany), according to the instructions of the manufacturer, using IDEALTM 96 extraction robot (Innovative Diagnostics, Montpellier, France). Then, CCHFV simplex reverse transcription (RT) quantitative PCRs were run, adapted from previously published studies [11,12]. Optimisations, including modifications to the PCR thermoprofile and mix, were carried out by Innovative Diagnostics, and allowed the quickest detection and the best amplification curve for a synthetic RNA used as positive control. The PCR detection limit was estimated to five viral genomic copies.

Positive tick homogenates as well as a set of negative ones were sent for confirmation to the Biosafety Level 4 laboratory of the National Reference Center for Viral Hemorrhagic Fevers (NRC), Lyon, France. After removing tick tissues from suspensions, RNA was extracted using the QIAamp viral RNA mini kit (Qiagen, Hilden, Germany) following the manufacturer’s instructions. Thereafter, detection of the virus was performed using the QIAGEN OneStep RT-PCR kit (Qiagen), according to a protocol adapted from Wölfel et al. [12].

The NRC selected samples with quantification cycle (Cq) values < 25 for whole genome sequencing. The RNA was subjected to a DNase digest (Turbo DNase, Thermo Fisher Scientific, Waltham, the United States (US)). Then, library preparation was performed using the NEBNext Ultra II RNA Library Prep Kit for Illumina (New England Biolabs, Ipswich, US). Sequencing was performed using a MiniSeq platform (Illumina Inc, San Diego, US). The paired-end reads generated were trimmed and de-novo assembled using rnaviralSPAdes (version 3.15.4) on Galaxy platform (version 23.2.rc1) (https://usegalaxy.org/). The CCHFV genomes (segment S, M and L) were searched from the contigs using Basic Local Alignment Search Tool (BLAST) (version 2.14.1). We used the BioEdit software (version 7.1.3.0) (https://bioedit.software.informer.com/) to align the nucleotide sequences obtained with published sequences belonging to the different lineages of CCHFV.

## Findings of ticks and Crimean–Congo haemorrhagic fever virus

In 2022, an average of 30 *H. marginatum* ticks per location, and in 2023 all ticks, were analysed for CCHFV.

In 2022, ticks were collected on 33 horse and three cattle farms. In total, 997 ticks of *H. marginatum* were identified and CCHFV was detected in 13 (1.3%) ticks (all from the same cattle farm in Pyrénées-Orientales) (Figure 1). In 2023, in Pyrénées-Orientales, ticks were collected on 15 cattle farms, three farms with cattle and horses and three farms with horses. A total of 1,001 *H. marginatum* ticks were collected and analysed, and 142 (14.2%) were positive for CCHFV. Most of these positive ticks were collected from cattle farms (n = 11), except for 12 ticks from two horse farms (Figure 1).

**Figure 2:**
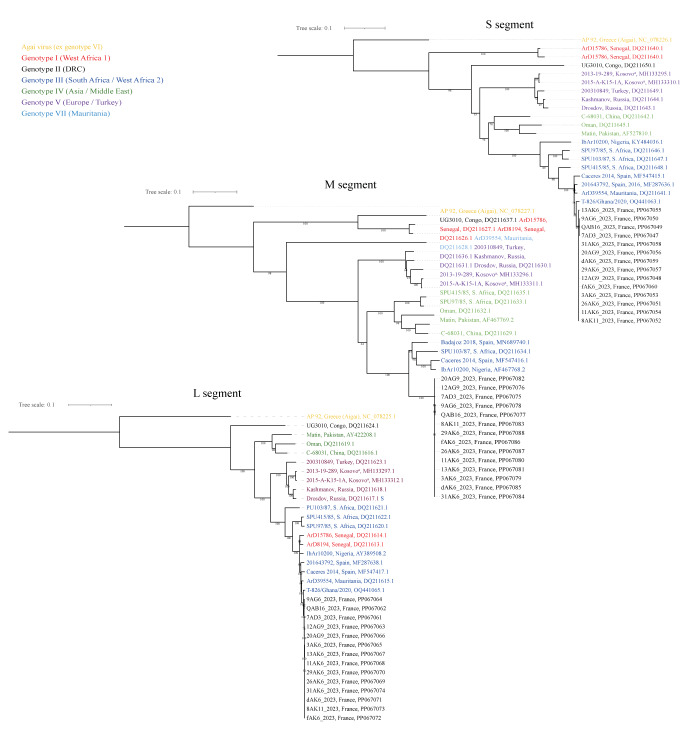
Phylogenetic analysis of Crimean–Congo haemorrhagic fever virus S, M and L segment sequences, France obtained in May 2022 and April 2023 and other countries

^a^ This designation is without prejudice to positions on status and is in line with United Nations Security Council Resolution 1244/99 and the International Court of Justice Opinion on the Kosovo Declaration of Independence.

Considering 2022 and 2023 data, the proportion of infected ticks in positive farms varied from 3.1% to 55.8% (median: 7.4%) or 1 to 66 positive ticks per farm, with four farms with infection rates up to 20%. The Cq values ranged from 18.25 to 40.82 (median:  36.39), which suggested a high viral load in some ticks. At the NRC, CCHFV RNA was confirmed in 132 (85.2%) of 155 samples, and six additional ones among the 114 initially negative samples. All Cq values from the 23 non-confirmed positive samples were high (around 40). All obtained sequences clustered with CCHFV strains, such as the Caceres strain previously isolated from ticks in Spain, within the genotype III (Figure 2).

The analysis was performed by using IQ-TREE software (version 2.1.2) (http://www.iqtree.org/).

Bootstrap values (> 50%) are shown at nodes. Scale bar represents the estimated number of substitutions per site. Individual sequences are named with strain name, country of origin and GenBank (https://www.ncbi.nlm.nih.gov/genbank/) accession number, if available. Each colour represents a genotype already described. All phylogenetic analyses were conducted on the complete coding DNA sequence (nucleoprotein for the S segment and RNA-dependent RNA polymerase for the L segment) except for the M segment for which partial sequences were obtained (from Nairovirus M polyprotein-like region to codon STOP).

## Discussion

For the first time, CCHFV was detected in resident tick populations in the mainland of France, confirming its circulation. Crimean–Congo haemorrhagic fever is one of the most widespread viral tick-borne zoonoses worldwide with severe consequences for healthcare, such as intensive case management, 5–30% fatality rate in haemorrhagic patients and the need to prevent nosocomial infections [13]. Endemic in Africa, the Middle East and other Asian countries and in the Balkans, CCHF was recently detected in Spain, western Europe [14]. Transmission of the virus to humans occurs predominantly via tick bites or via exposure to infected blood or tissues of viraemic animals or patients [1]. Given the recent findings of *H. marginatum,* one main competent vector for CCHFV, in the south of France [8,15] near the Spanish border, it was crucial to evaluate the epidemiological situation of CCHFV in France.

We considered that CCHFV circulation in France would be at a low level. Indeed, immature stages of *H. marginatum* are mainly found on birds and lagomorphs. Most bird species are refractory to CCHFV and thus unable to reinfect naïve ticks, whereas lagomorphs are good amplifiers of the virus. In France, bird populations are sizeable in the south of the country. The populations of hares and rabbits have decreased in France since the outbreak of myxomatosis in the 1950s and the current occurrence of rabbit viral haemorrhagic disease. Consequently, the probability for *H. marginatum* to become infected at the immature tick stage in France is considered low [7]. Furthermore, adult stages of *H. marginatum* have a marked trophic preference for horses in France [5], and horses are poor amplifiers of CCHFV (unable to reinfect new ticks) although they are very abundant in the south of France. Thus, the probability for *H. marginatum* to become infected at the adult stage is considered low [7].

Our findings of a high proportion of infected ticks in some farms further reinforced that this cannot be due to sporadic introduction of infected *H. marginatum* ticks from endemic countries but local virus transmission. However, such introduction events remain a risk to be monitored, especially to detect the emergence of new CCHFV genotypes [16-18]. All CCHFV isolates sequenced in this study were highly identical and belonged to the same genotype. Ticks infected with CCHFV were predominantly from cattle although many horses were examined, with high proportions of infected ticks in four facilities where very few animals were examined (4–6 animals per farm). This strengthens the importance of cattle in the CCHFV transmission and the possibility of having sampled viraemic animals. As horses are not able to replicate sufficiently CCHFV to infect naïve ticks [19], finding a few infected ticks on horses indicated that these *H. marginatum* were infected at immature stages or through transovarial transmission, and then maintained the virus along their development.

Because of the risk-based sampling method used, the proportion of infected ticks does not reflect the prevalence of *H. marginatum* ticks infected with CCHFV. The prevalence of infected ticks is presumably lower which may explain that no human cases have, so far, been notified in France. Additionally, genotype III, which was identified from the first Spanish cases [20], is considered of low virulence. At present, in France, humans may become infected via bites of adult *H. marginatum* ticks. This risk exists during the activity period of the ticks, namely from April to July. Only ticks seeking hosts in the environment should be considered as possible vehicles for virus transmission; those already attached to animals cannot detach and reattach. Risk areas for CCHFV transmission present conditions conducive to the presence of *H. marginatum* (open dry natural environments, typical of the Mediterranean area), but infected ticks were not found in all locations infested by *H. marginatum* and further parameters suitable for local circulation of CCHFV between ticks and animals, remain to be determined. Another CCHFV transmission pathway could be contact with infected animal material, as animals can be viraemic, albeit asymptomatic, during the seasonal activity of *H. marginatum* e.g. from April to July when adult stages parasite wild and domestic ungulates and from August to October when immature stage parasite lagomorphs. Considering such risks, preventive recommendations towards at-risk populations are needed.
